# Association between dietary intake of selenium and chronic kidney disease in US adults: a cross-sectional study of NHANES 2015–2018

**DOI:** 10.3389/fnut.2024.1396470

**Published:** 2024-08-13

**Authors:** Ying Pi, Xianyong Liao, Xiaodan Song, Yuyu Cao, Xiaona Tang, Guobing Lin, Yanghong Zhong

**Affiliations:** ^1^Shenzhen Bao'an District Traditional Chinese Medicine Hospital, Shenzhen, China; ^2^Shenzhen Traditional Chinese Medicine Hospital, Shenzhen, China

**Keywords:** chronic kidney disease, dietary selenium, NHANES, US adults, cross-sectional study

## Abstract

**Background:**

Chronic kidney disease (CKD) is currently a widespread chronic illness, and its development is influenced by nutrients. Selenium plays a crucial role in the intervention and therapy of various chronic illness. In this study, we aimed to investigate the connection between dietary selenium intake and CKD in adults in the United States.

**Methods:**

We included 6,390 individuals from the datasets of the National Health and Nutrition Examination Survey (NHANES) between 2015 and 2018. We used multiple logistic regression, restricted cubic spline regression, and forest plots to investigate the connection between dietary selenium intake and CKD.

**Results:**

After fully adjusting the data of 6,390 individuals from NHANES between 2015 and 2018, 1,523 (23.83%) of the individuals were identified as having chronic kidney disease (CKD). The rates of CKD in participants with average selenium intakes of ≤0.072, 0.072–0.103, 0.103–0.144, and > 0.144 mg/day were 27.53, 25.11, 22.42, and 19.96%, respectively. After adjusting for potential confounding factors, the fully adjusted odds ratio (OR) values for CKD according to dietary selenium intake were 1 (reference), 0.94 (95% confidence interval (CI): 0.79–1.12, *p* = 0.466), 0.82 (95% CI:0.68–0.98, *p* = 0.033), and 0.77 (95% CI:0.63–0.95, *p* = 0.016) for the four selenium intake levels, respectively, with P trend = 0.007. The dietary selenium intake was negatively associated with the incidence of CKD, after adjusting for other confounding factors. The risk of CKD decreased by 7.7% for every additional 0.1 mg of dietary selenium intake.

**Conclusion:**

A higher dietary selenium intake correlates significantly and negatively with the incidence of CKD.

## Introduction

Chronic kidney disease (CKD) describes a cluster of conditions resulting from various factors that cause the kidneys to have abnormal structure or function for a duration exceeding 3 months. According to relevant research reports (1, 2), the global prevalence of CKD is about 9.1%. Chronic kidney disease has induced a heavy burden on the global public health system. Currently, the prevalence of CKD in the US health insurance system has reached 14.5% (3, 4). The development of CKD is intimately linked to genetic factors, environmental factors, and nutritional factors (5–7). Current research indicates that trace elements play a pivotal role in preventing and treating various diseases (8). Studies have also found that CKD is closely related to nutrition and diet (9, 10). Therefore, identifying these controllable factors, such as diet and the intake of natural compounds, might help to prevent and treat CKD.

Selenium is crucial for human and animal life activities, affecting many physiological functions of the human body, and playing a key role in anti-inflammatory and antioxidant processes (11). Humans cannot synthesize selenium, which must be obtained from external intake. Selenium is mainly enriched in the human kidney, where it can be used to synthesize various selenoproteins. The essential role of selenium in the human body is primarily fulfilled by 25 selenoproteins, with selenocysteine serving as the active component (12, 13). These selenoproteins function as inhibitors of non-specific immune responses and inflammatory reactions, as well as factors affecting reproduction and oxidative stress. During the recovery period after injury, some selenoproteins (such as glutathione peroxidase 4 (GPX4), GPX1, selenoprotein P, and selenoprotein S) can interact to produce numerous anti-inflammatory and antioxidant activities, and can reduce the production of certain superoxide radicals that cause inflammatory reactions (14, 15). Previous studies have shown that the serum selenium content of patients with CKD is lower than that of healthy people, and selenium deficiency is closely related to the occurrence of CKD. and the decrease is more pronounced in patients receiving hemodialysis (15, 16). Studies have shown that selenium administered orally or by intravenous injection can improve the immune function of patients with CKD and reduce oxidative stress-induced kidney damage (17–19). Therefore, appropriate selenium supplementation might help to reduce the risk of CKD.

Currently, most selenium-containing drugs have different limitations and side effects, such as causing gastroenteritis, acropachy, and cognitive function decline (20). Therefore, increased research attention has been paid to dietary methods of selenium supplementation. Many natural compounds, such as phosphorus, vitamin D, magnesium and other nutrients have also been shown to have a certain preventive and therapeutic effect on CKD (21–23). However, according to our investigation, there is no study directly discussing the correlation between dietary selenium and CKD at present.

Therefore, in this study, we used 4 years of NHANES data (2015–2018) to investigate the relationship between dietary selenium intake and CKD. In addition, we explored whether this relationship varied among individuals according to age, sex, weight, and disease status. We hypothesized that a higher selenium intake is inversely associated with the incidence of CKD.

## Methods

### Study population

The data of this study were obtained from the US National Health and Nutrition Examination Survey (24). The NHANES is a nationally representative survey of the non-institutionalized civilian population in the United States, conducted by the Centers for Disease Control and Prevention (CDC). It includes information from many personal interviews, dietary intake surveys, laboratory tests, and other health-related questions. The data used in this study were collected and organized by the National Center for Health Statistics (NCHS). The data from NHANES are publicly available and have been used to explore the relationship between nutrients and certain diseases in the general population (25).

**Figure 1:**
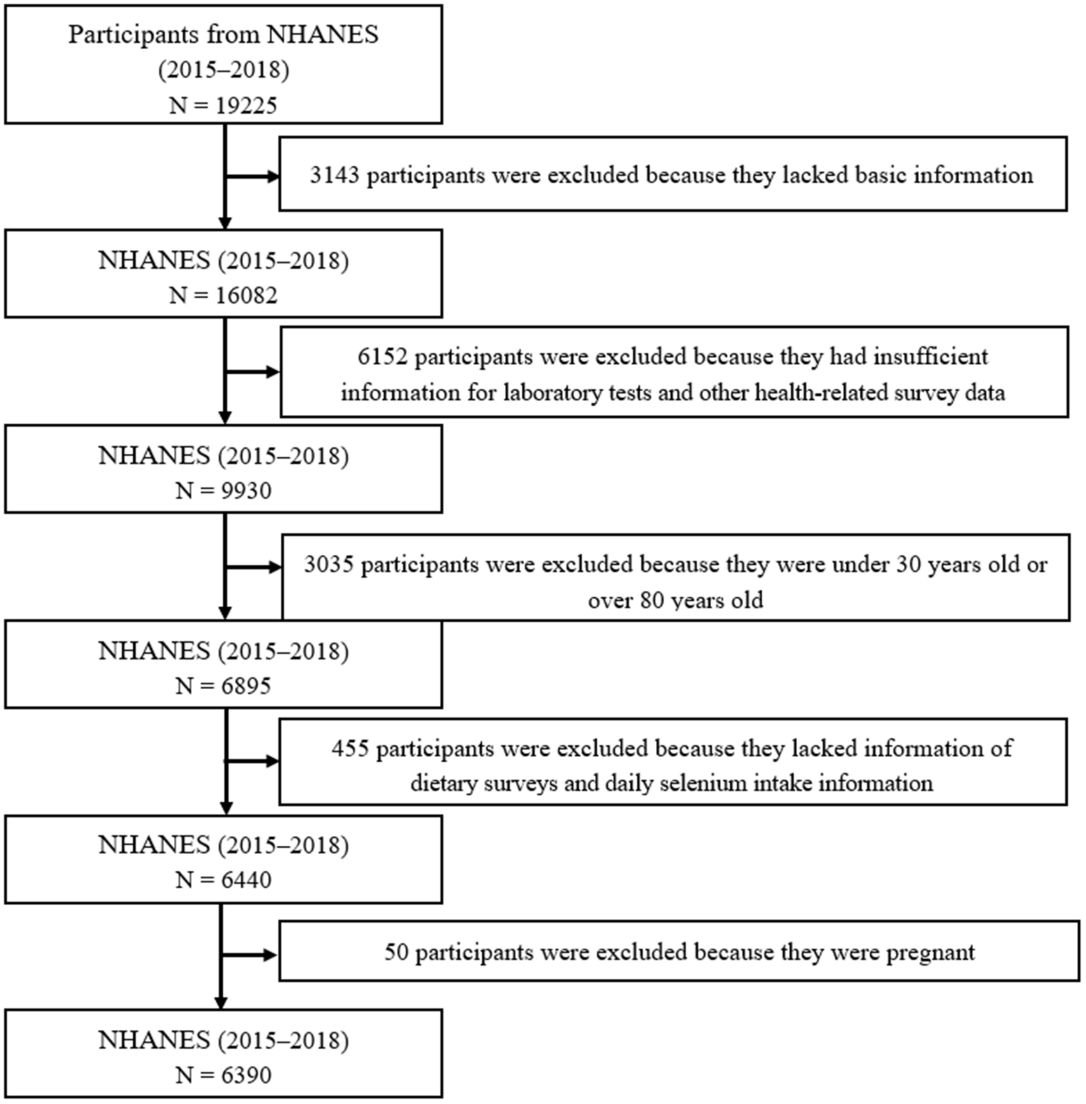
Flowchart of the study population. NHANES, National Health and Nutrition Examination Survey.

From the NHANES data from 2015–2018, a total of 19,225 participants were surveyed for CKD. The inclusion criteria are as follows: (1) Participants aged 30 to 80 years old; (2) Participants who participated in the renal function blood and urine subgroup study; (3) Participants’ chronic kidney disease (CKD) status information was confirmed based on the US NHANES questionnaire data. The exclusion criteria are as follows: (1) Participants lacking basic information(*N*=3143); (2) Participants who have insufficient information for laboratory tests and other health-related survey data(*N*=6152); (3) Participants whose age under 30 years old or over 80 years old (*N*=3035); (4) Participants who lacking information of dietary surveys and daily selenium intake information (*N*=455); (5) Participants who are pregnant women (*N*=50) according to the USA NHANES questionnaire. Finally, 6,390 adults (3,131 men and 3,259 women) were included in this study (Figure 1).

### Diagnosis of chronic kidney disease and dietary assessment

The serum creatinine was converted to the estimated glomerular filtration rate (eGFR) according to the CKD-Modification of Diet in Renal Disease (CKD-MDRD) equation calculation formula published in the Journal of the American Society of Nephrology in 2006 (26). If the eGFR is <60 mL/ (min/1.73 m^2^), or albuminuria (urine albumin to creatinine ratio (ACR) ≥ 30 mg/g) is present, then CKD is diagnosed. The international standard unit for the serum creatinine level is μmol/L, and we used the serum creatinine correction formula recommended in the NHANES III database documentation for the calculation (27). The NHANES dietary survey records refer to the data of all beverages and dietary intakes of the respondents within 1 day. From 2015 to 2018, the Automated Multiple-Pass Method (AMPM) of the United States Department of Agriculture (USDA) was used to collect the respondents’ dietary records. The AMPM collects detailed information about each person’s food and converts it into various nutrient contents (28). Therefore, while the 24-h dietary recall record might have certain limitations in terms of validity and reliability in nutritional assessment, it has certain advantages in providing information on food types and quantities.

### Assessment of covariates

The data were obtained from the NHANES database from 2015 to 2018. The demographic characteristics of the covariates in this study included sex, age, race, family poverty income ratio, marital status, education level, body mass index (BMI), lifestyle and diet, (including smoking, and carbohydrate intake), C-reactive protein, triglycerides, serum uric acid, and total cholesterol (from the laboratory tests in the NHANES database). Smoking status was categorized as smokers (having smoked more than 100 cigarettes throughout their lifetime) and non-smokers (having smoked less than 100 cigarettes throughout their lifetime). Disease history included diabetes (diagnosed based on doctor’s assessment and self-reported diabetes) and hypertension (diagnosed mainly based on doctor’s notification of hypertension and systolic blood pressure ≥ 140 mmHg or diastolic blood pressure ≥ 90 mmHg).

### Statistical analyses

Statistical analysis was performed using IBM SPSS software (version 25.0; IBM Corp., Armonk, NY, United States). In this study, continuous and categorical variables were expressed as mean (standard deviation, SD), and frequency variables were expressed as percentages. The comparison of continuous and categorical variables was performed employing the chi-squared test, and the frequency variable was tested using an independent sample T test to evaluate the differences between patients with CKD and healthy respondents. We categorized the individuals into four groups based on the selenium content in their diet, which were divided into quartiles. Multiple logistic regression analysis was utilized to investigate the association between dietary selenium intake and CKD, and the data are presented as odds ratios (ORs) with 95% confidence intervals (CIs). A *p*-value less than 0.05 was regarded as statistically meaningful. The association between dietary selenium intake and CKD was investigated using restricted cubic spline analysis. In model 1, we adjusted the model based on variables such as age, sex, and race. In model 2, we used the same variables as in model 1, with further adjustments.

## Results

### Baseline characteristics

Table 1 presents the basic characteristics of the 6,390 individuals who had complete information from the NHANES data between 2015 and 2018. Among these respondents, 1,523 (23.8%) were patients with CKD. Compared with the healthy people, patients with CKD were more likely to be older, non-Hispanic whites, smokers, highly educated, economically disadvantaged, and have diabetes, hypertension, a higher BMI, higher blood pressure level, higher total cholesterol levels, higher uric acid levels, higher carbohydrate intake, and lower selenium intake (Table 1).

**Table 1:** Characteristics of participants with or without CKD.

Characteristics	Non-CKD	CKD	*p* value
Participants, No. (%)	4,867 (76.2)	1,523 (23.8)	
**Sex, No. (%)**			
Male	2,385 (49.0)	746 (49.0)	0.988
Female	2,482 (51.0)	777 (51.0)	
Age, Mean (SD), years	51.86 ± 13.93	63.85 ± 13.60	<0.001
**Race/ethnicity, No. (%)**			
Mexican American	714 (14.7)	181 (11.9)	<0.001
Non-Hispanic white	1,435 (29.5)	281 (18.5)	
Non-Hispanic black	1824 (37.5)	638 (41.9)	
Others	894 (18.4)	423 (27.8)	
**Marital status No. (%)**			
Married or living with partner	3,224 (66.2)	855 (56.1)	<0.001
Living alone	1,643 (33.7)	668 (43.9)	
**Education level No. (%), years**			
<High School	928 (19.1)	332 (21.8)	<0.001
High school or GED	1,078 (22.2)	389 (25.5)	
>High School	2,861 (58.8)	802 (52.7)	
**Smoking No. (%)**			
Smoker	2,164 (44.5)	750 (49.2)	0.004
Never Smoker	2,703 (55.5)	773 (50.8)	<0.001
Ratio of family income to poverty	2.69 ± 1.62	2.39 ± 1.53	<0.001
Diabetes No. (%)	644 (13.2)	529 (34.7)	<0.001
Hypertension No. (%)	1708 (35.1)	998 (65.5)	<0.001
Selenium supplement No. (%)	490 (10.1)	182 (12.0)	0.065
BMI, Mean (SD), kg/m^2^	28.74 ± 6.58	29.59 ± 6.84	<0.001
Carbohydrate intake (g/day)	249.60 ± 121.46	233.28 ± 121.66	<0.001
HS C-Reactive protein (mg/dL)	0.39 ± 0.65	0.57 ± 1.17	<0.001
Triglycerides(mmol/L)	1.75 ± 1.41	1.90 ± 1.63	0.001
Uric acid (μmol/L)	315.17 ± 81.25	357.89 ± 98.35	<0.001
Total Cholesterol (mmol/L)	5.00 ± 1.04	4.87 ± 1.18	<0.001
ACR (mg/g)	8.66 ± 5.73	175.39 ± 588.18	<0.001
Selenium intake (mg/day)	0.12 ± 0.07	0.11 ± 0.06	<0.001

Body mass index (BMI) was calculated as BMI = weight/height^2^; SD, standard deviation.

CKD, chronic kidney disease; GED, General Educational Development; HS, high sensitivity; ACR, albumin to creatinine ratio.

### Association between dietary intake of selenium and CKD

**Figure 2:**
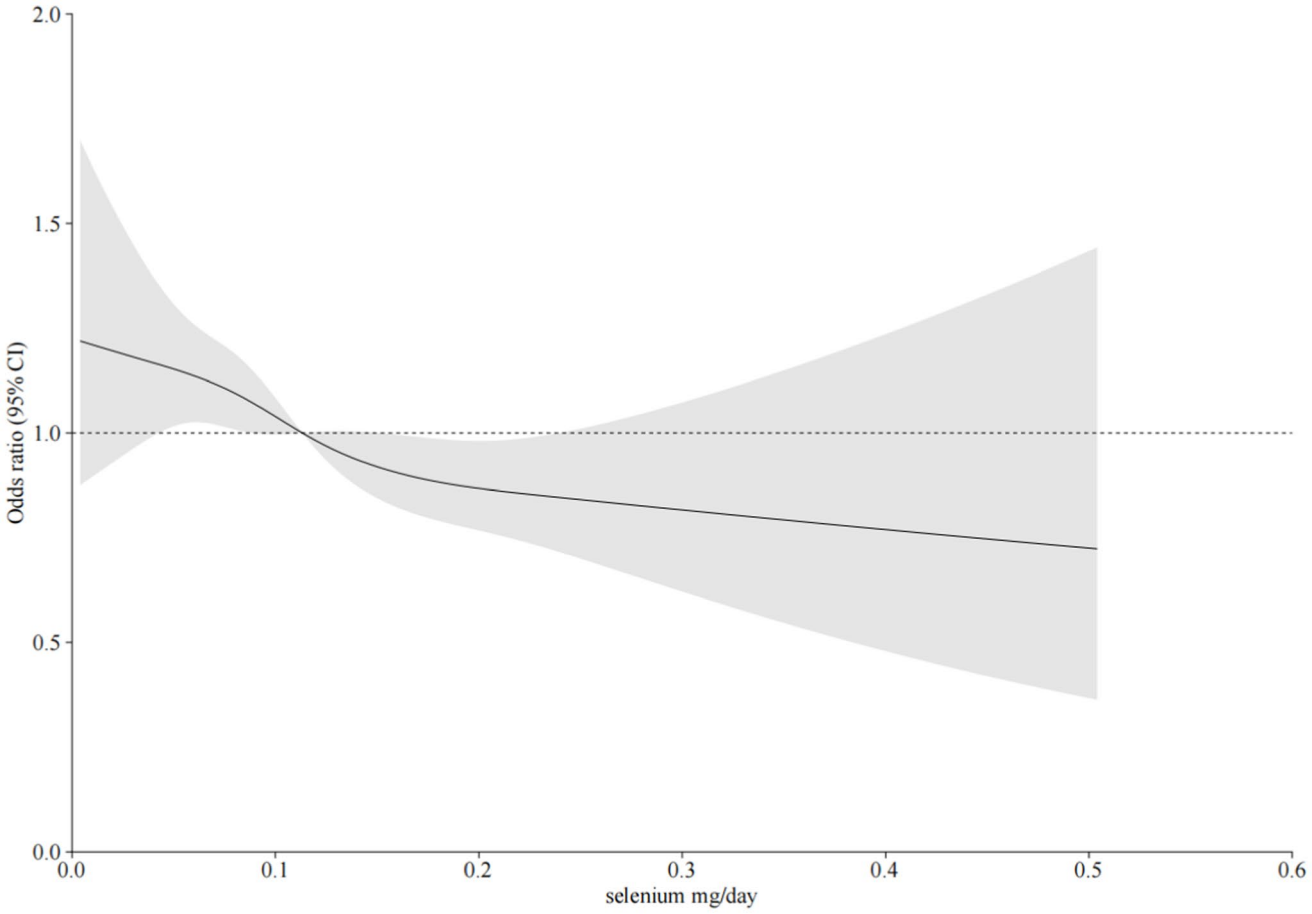
The relationship between dietary selenium intake and CKD was investigated using restricted cubic spline regression (RCS). The model was adjusted for sex, age, poverty ratio, race/ethnicity, marital status, education level, smoking, body mass index (BMI), hypertension, diabetes, C reactive protein, total cholesterol, triglycerides, uric acid, carbohydrate intake, and selenium supplement intake. Solid line, odds ratio (OR); shade, 95% confidence interval (CI).

As shown in Table 2, after adjusting for potential confounding factors, there was a significant negative correlation between daily dietary selenium intake and the incidence of CKD. The daily selenium intake was divided into quartiles Q1 (≤ 0.072 mg/day), Q2 (0.072–0.103 mg/day), Q3 (0.103–0.144 mg/day), and Q4 (> 0.144 mg/day) according to the quartiles. The odds ratios (ORs [95% CI]) for the connection between dietary selenium intake and CKD prevalence across the four quartiles were as follows: 1 (reference), 0.94 (0.79–1.12), 0.82 (0.68–0.98), and 0.77 (0.63–0.95) (P trend = 0.007), respectively. And the odds ratios for the relationship between each standard deviation of selenium intake and the prevalence of CKD were 0.85 (0.80-0.91), 0.93 (0.81-1.05), and 0.93 (0.83-1.09), respectively. According to the results of restricted cubic spline analysis, there was a significant negative correlation between dietary selenium intake levels and the prevalence of CKD (Figure 2).

**Table 2:** Association between dietary selenium and CKD.

Dietary selenium (mg/day)	No.	Crude OR (95% CI)	*p*-value	Mode 1 OR (95% CI)	*p*-value	Mode 2 OR (95% CI)	*p*-value
	1523/6390	0.09 (0.03–0.24)	<0.001	0.23 (0.08–0.67)	0.007	0.23 (0.07–0.76)	0.017
Per-SD		0.85 (0.80-0.91)	<0.001	0.93 (0.81-1.05)	0.025	0.93 (0.83-1.09)	0.059
Q1	454/1649	1 (Ref)		1 (Ref)		1 (Ref)	
Q2	407/1621	0.88 (0.76–1.03)	0.116	0.89 (0.75–1.05)	0.177	0.94 (0.79–1.12)	0.466
Q3	357/1592	0.76 (0.65–0.89)	0.001	0.81 (0.68–0.96)	0.017	0.82 (0.68–0.98)	0.033
Q4	305/1528	0.66 (0.56–0.78)	<0.001	0.77 (0.64–0.92)	0.004	0.77 (0.63–0.95)	0.016
*P-*trend		<0.001		0.002		0.007	

Q, quintile, the daily selenium intake was divided into Q1 (≤ 0.072 mg/day), Q2 (0.072–0.103 mg/day), Q3 (0.103–0.144 mg/day), and Q4 (> 0.144 mg/day); Ref, reference.

Model 1 was adjusted for age, sex, and race/ethnicity, marital, education level, body mass index (BMI), hypertension, and diabetes. OR, odds ratio; CI, confidence interval.

Model 2 was adjusted for all the variables in Model 1 plus dietary intake (carbohydrate intake, selenium supplement intake) and clinical characteristics (C reactive protein, total cholesterol, triglycerides, and uric acid).

**Figure 3:**
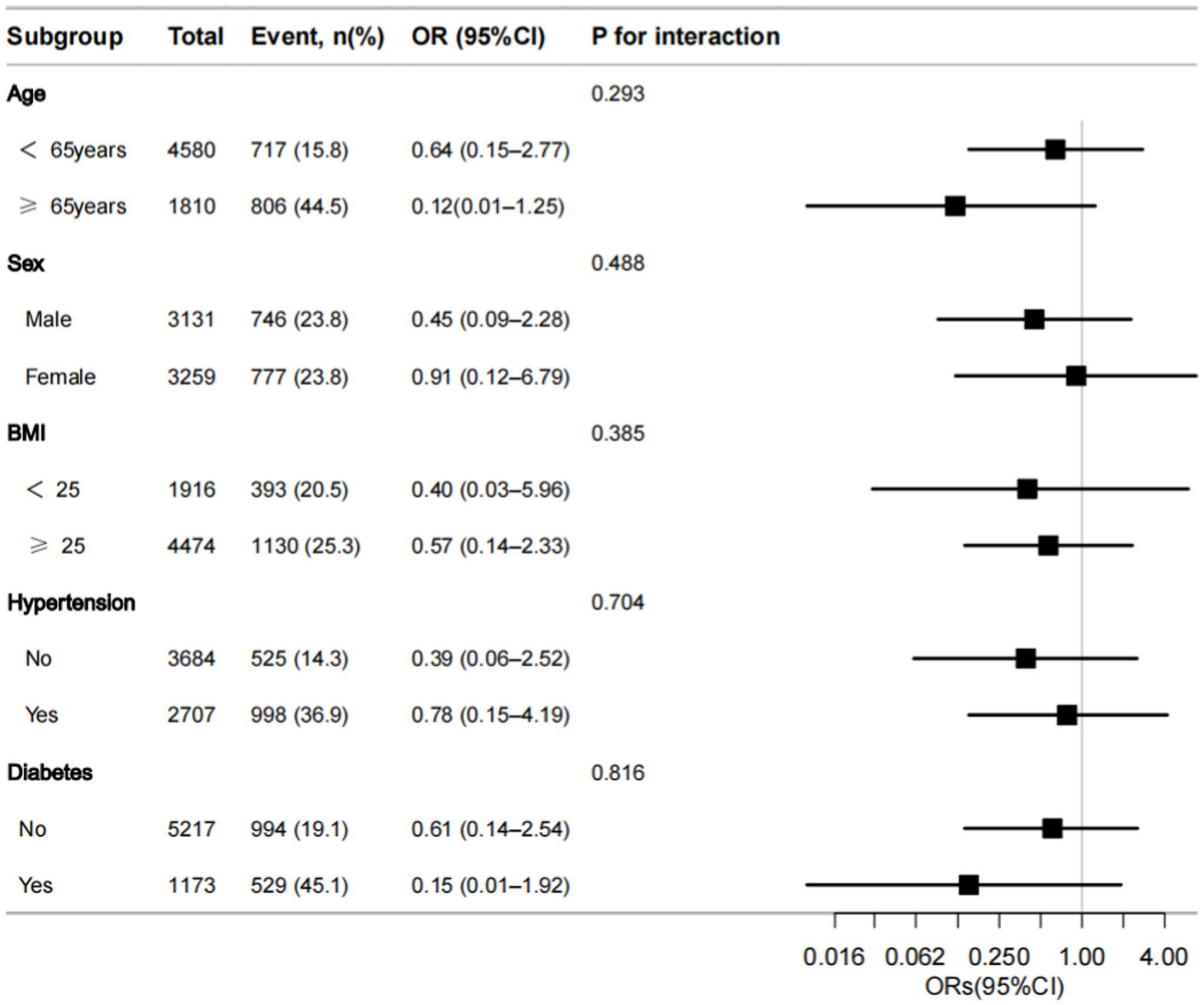
Forest plot of odds ratios (ORs) of dietary selenium intake with CKD in the subgroup analyses data. ORs are presented with 95% confidence intervals (95% CI). The limit lines represent the 95% CI. BMI, body mass index.

As shown in Figure 3, we undertook a subgroup analysis among the study population, taking into account potential confounding factors. According to the forest plot, the joint analysis results showed that after stratification based on age, sex, body weight, diabetes, and hypertension, it was found that there was still a negative correlation between dietary selenium intake and the incidence of CKD, and there was no individual difference due to these factors.

## Discussion

To the best of our knowledge, there have been few studies concerning the connection between dietary selenium and CKD. In this cross-sectional study, after adjusting for confounding factors such as sex, age, race, marital status, education level, smoking status, poverty rate, carbohydrate intake, BMI, diabetes, hypertension, C-reactive protein, triglycerides, total cholesterol, serum uric acid, and dietary selenium supplement intake, we confirmed the reliability of the significant negative correlation between dietary selenium intake and the incidence of CKD. In the population studied, we found that after adjusting for other confounding factors, the risk of CKD decreased by 7.7% for every additional 0.1 mg of dietary selenium intake.

The United States Food and Nutrition Board (FNB) has found that the demand for selenium increases with age, and it is recommended that people under the age of 30 consume 50–100 micrograms of selenium per day, while people over the age of 30 should consume 100–200 micrograms of selenium per day to help prevent genetic diseases and related chronic diseases (29–31). We took into account the different recommended doses for people over and under the age of 30, and primarily focused on examining the association between dietary selenium intake and CKD among individuals aged 30 and above in the United States.

Compared with other trace elements, selenium plays an important role in the human body, especially in anti-inflammatory, antioxidant, and immune regulation processes (32, 33). Currently, the association between selenium intake and CKD is controversial. In this study, we confirmed the negative correlation between selenium and the occurrence of CKD. Similarly, Xie et al. (34) found that among 5,381 middle-aged and elderly people in the China Health and Nutrition Survey (CHNS) database, the CKD prevalence was 23.33 and 9.25% in people with daily selenium intake levels of 21.5 ± 4.82 μg/day and 67.0 ± 13.97 μg/day, respectively. The occurrence of CKD was 2.5 times greater in the group with low selenium intake compared with that in the group with high selenium intake. A higher selenium intake might play a positive role in renal function. This finding is consistent with the results of the present study. Zhu et al. (35) studied 3,063 CKD patients in the NHANES database (during 1999–2000, 2003–2004, and 2011–2018) found that the serum selenium levels <156.8 μg/L and 201.5–734.8 μg/L had a 10-year mortality risk ratio (HRs; 95% CI) of 0.866 (0.724–1.035) and 0.684 (0.549–0.852) (P trend<0.001), respectively. However, the serum selenium content in CKD patients receiving dialysis treatment may be affected by blood dialysis, there may be survivor bias in analyzing the relationship between serum selenium levels and mortality risk in CKD patients. Compared with serum selenium, the dietary structure of the population is often more stable, so there are fewer factors that can interfere with the study of dietary selenium.

Fu et al. (36) conducted a Mendelian randomization analysis of 9,639 large-scale genome-wide association studies in Europe and found that the serum selenium level exhibits a negative correlation with the estimated glomerular filtration rate (eGFR) among European individuals (HR = −0.0042, 95% CI: −0.0053–0.0031, *p* = 2.186 × 10^−13^), which seems to be contrary to our results. We speculate that there are several possible explanations for this difference. First, dietary selenium intake levels and baseline serum selenium levels have distinct differences. Second, studies have shown that serum selenium levels are easily affected by oxidative stress status in the human body (37), while daily dietary conditions are less affected by oxidative stress status. Finally, the study population is different, and the results may be affected by geographical, ethnic, genetic, and other relevant factors.

Some animal clinical trials have also found that dietary intake or oral selenium supplements can alleviate kidney injury and renal fibrosis to a certain extent in rats, thereby improving renal function (38, 39). Based on this, we analyzed the relationship between dietary selenium intake and CKD patients. This may provide a theoretical basis for future researchers to conduct clinical controlled trials of selenium and CKD.

The biological mechanism between dietary selenium intake and CKD is currently unclear but it might be linked to the following mechanisms. Selenium can reduce oxidative stress injury by regulating immune responses, inhibiting inflammatory factor release, and downregulating TGF-β1 expression of cytokines to reduce kidney damage and improve renal function in patients (14, 33, 40). Selenium is an important component of several antioxidant enzyme substances and can also enhance the antioxidant effects of non-enzyme substances such as vitamin E and coenzyme Q-10. Therefore, selenium can reduce oxidative stress reactions in the human body to a certain extent and protect the kidneys (12). Finally, our study results supported the positive role of selenium in preventing CKD.

Since our current research aims to verify or explore a specific hypothesis or theory, The formula for converting serum creatinine in the CKD-Dietary Modification of Renal Disease (CKD-MDRD) study into estimated glomerular filtration rate (eGFR) has undergone long-term validation, comparison with the latest formula this formula has higher authority. Therefore, we chose the CKD-MDRD equation, published in the Journal of the American Society of Nephrology in 2006, to calculate the glomerular filtration rate when assessing the renal function of the included population.

## Limitation section

There are some advantages in our current study. As far as we know, this is the initial study to examine the relationship between dietary selenium intake and CKD among American adults. Additionally, we have also evaluated the correlation between selenium intake and CKD, and provided some practical suggestions. However, there are also some disadvantages in this study. Firstly, our findings are specific to the American adult population and may not be generalized to other global populations. Secondly, in this study, we diagnosed CKD based on serum creatinine levels and proteinuria, the NHANES database lacks kidney imaging data, and we may miss some patients with CKD. Therefore, to some extent, we may have missed some CKD patients. Future studies can appropriately incorporate renal imaging results for diagnosis if possible. Thirdly, we only studied the sample data of American adults from 2015 to 2018. Future studies can conduct more extensive and larger sample size studies. At last, our current study is a cross-sectional investigation, and as such, it cannot establish a valid cause-and-effect relationship. Therefore, in future studies, we need to conduct prospective longitudinal studies to further confirm the relationship between selenium and CKD.

## Conclusion

In conclusion, through our study, we found that the daily dietary selenium intake of American adults is significantly negatively correlated with the incidence of CKD.

## Data availability statement

The datasets presented in this study can be found in online repositories. The names of the repository/repositories and accession number(s) can be found at: http://www.cdc.gov/nchs/nhanes.htm.

## Ethics statement

The research conducted in this study involved the analysis of a nationally representative public dataset that has been openly published online and is accessible for download and analysis by anyone. The studies were conducted in accordance with the local legislation and institutional requirements. The human samples used in this study were acquired from the blood samples were provided by the NHANES database. Written informed consent for participation was not required from the participants or the participants' legal guardians/next of kin in accordance with the national legislation and institutional requirements.

## Author contributions

YP: Writing – original draft, Formal analysis. XL: Writing – original draft. XS: Validation, Writing – review & editing. YC: Writing – review & editing, Validation. XT: Writing – review & editing, Formal analysis. GL: Writing – review & editing, Conceptualization. YZ: Writing – review & editing, Conceptualization.
